# Supplementary material for: Rapamycin-Induced Feedback Activation of eIF4E-EIF4A Dependent mRNA Translation in Pancreatic Cancer
Source: Cancers (Basel). 2023 Feb 24;15(5):1444. doi: 10.3390/cancers15051444 (PMC10001351; doi:10.3390/cancers15051444)
Supplement: Supplementary file 1 [file cancers-15-01444-s001.zip › Supplementary_Figures.pdf]

Supplementary Figures Legends:

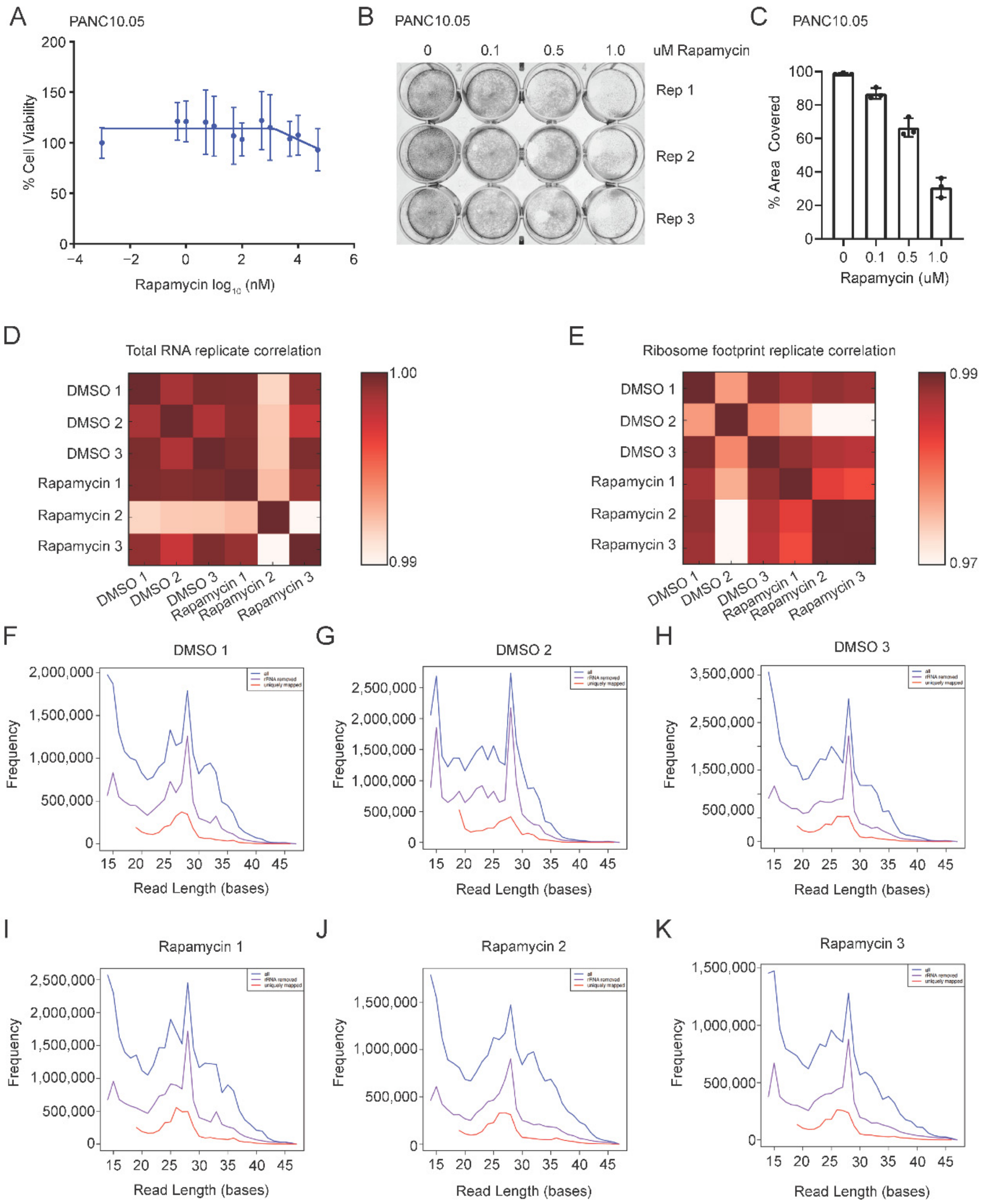

Supplementary Figure S1. Quality control analysis of the ribosome footprinting data. **A.** Cell viability assay showing the IC50 for rapamycin in PANC10.05 cells. **B and C.** Clonogenic assay shows that rapamycin treatment inhibits cell growth in PANC10.05

cells. **D and E.** Read count correlation plots of replicates from DMSO and rapamycin-treated total RNA **(D)** and ribosome footprinting **(E)** samples. **F-K.** Frequency distribution of the read length distribution in replicates from DMSO **(F-H)** and rapamycin-treated **(I-K)**.

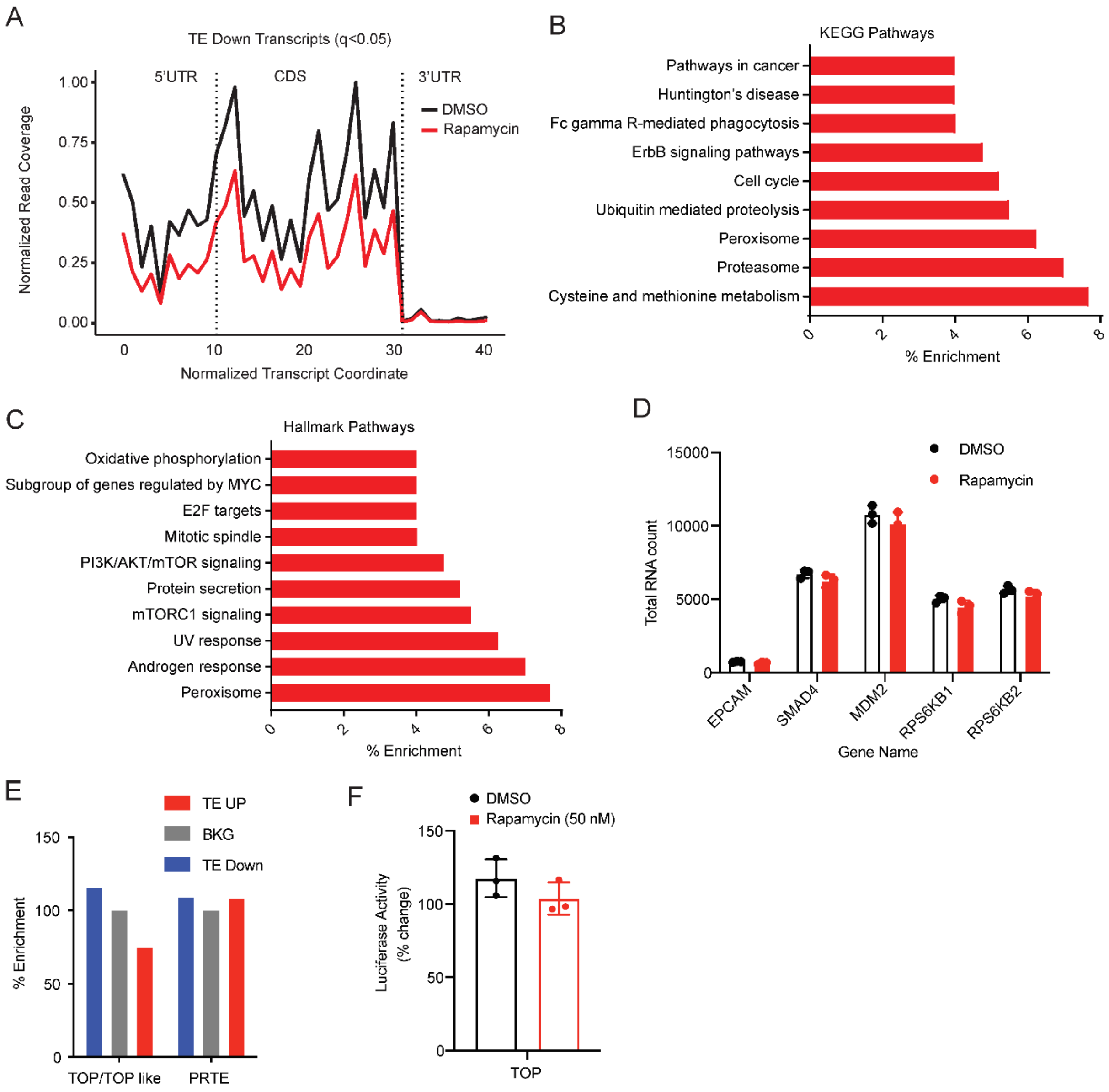

Supplementary Figure S2. Rapamycin inhibits the translation of genes involved in the cell cycle and cancer cell growth. **A.** Metagene plot showing that the ribosome coverage

is reduced throughout the mRNA length in the TE down mRNAs (q<0.05) in rapamycin-treated PANC-1 cells compared to the DMSO. RF coverage and transcript length are normalized. **B.** GSEA KEGG pathway analysis of TE down genes (q<0.05) in rapamycin-treated PANC-1 cells. **C.** GSEA Hallmark pathway analysis of TE down genes (q<0.05) in rapamycin-treated PANC-1 cells. **D.** Total mRNA expression of indicated TE down targets in rapamycin-treated PANC-1 cells compared to DMSO controls. **E.** TOP/TOP-like and PRTE motif is enriched in TE down genes. **F.** Rapamycin inhibits TOP motif-dependent translation as observed by luciferase reporter assay. TOP motif luciferase activity is normalized to cap-dependent activity.

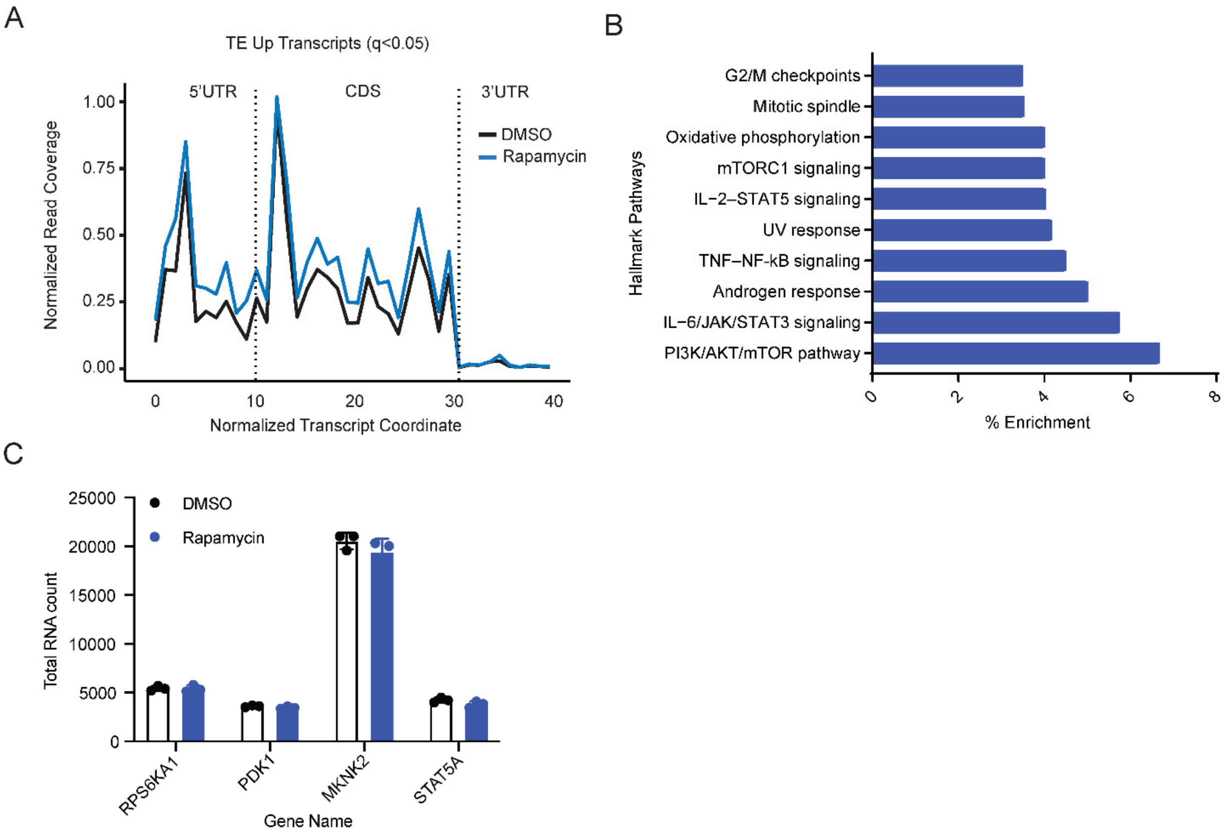

Supplementary Figure S3. Rapamycin induces the translation of a subset of mRNAs. **A.** Metagene plot showing that the ribosome coverage is increased throughout the mRNA

length in the TE up mRNAs (q<0.05) in rapamycin-treated PANC-1 cells compared to the DMSO. RF coverage and transcript length are normalized. **B.** GSEA KEGG pathway analysis of TE up genes (q<0.05) in rapamycin-treated PANC-1 cells. **C.** Total mRNA expression of indicated TE up targets in rapamycin-treated PANC-1 cells compared to DMSO controls.

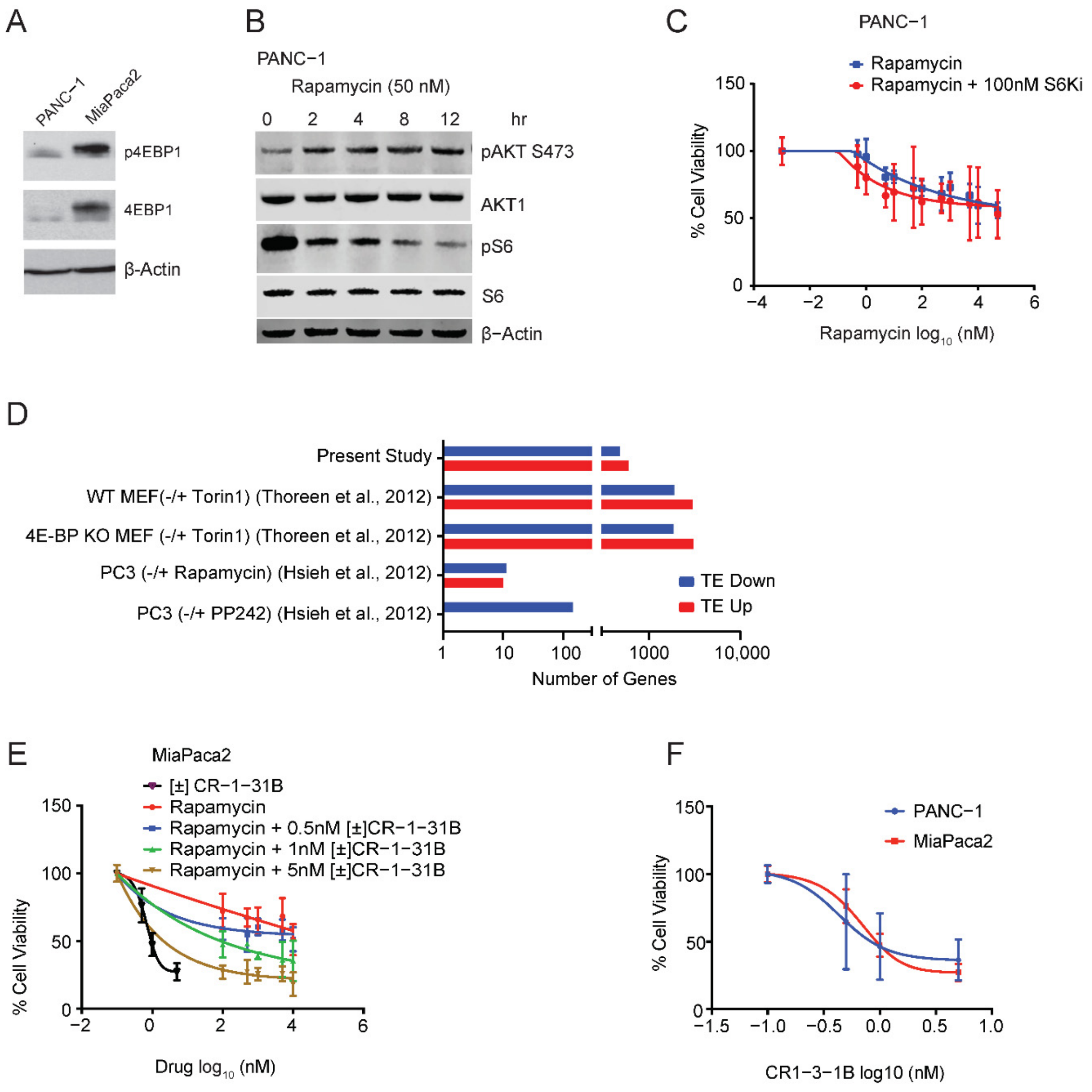

Supplementary Figure S4. Rapamycin activates translation through feedback activation of AKT1 and eIF4E. **A.** Immunoblot showing the expression of phospho-4EBP1 and total

4EBP1 in PANC-1 and MiaPaca2 cells. β-actin is used as the loading control. **B.** Immunoblot showing the downregulation of phospho-S6 and activation of phosphor-AKT1 following rapamycin treatment (50 nM) for the indicated time points in PANC-1 cells. Total S6 remains unaffected. β-actin is used as the loading control. **C.** Cell viability assay showing that combination of S6Ki (100 nM) has no significant effect on the anti-proliferative effect of rapamycin in PANC-1 cells. **D.** Number of genes regulated by Rapamycin as detected in the present study and the previous studies from Thoreen et al, 2012 and Hseih et al, 2012. **E.** Cell viability assay showing that the combination of CR-1-31B enhances the anti-proliferative effect of rapamycin in MiaPaca2 cells. **F.** Cell viability assay showing that IC50 for CR-1-31B alone in PANC-1 and MiaPaca2 cells.

A. Full blots corresponding to Fig 2H

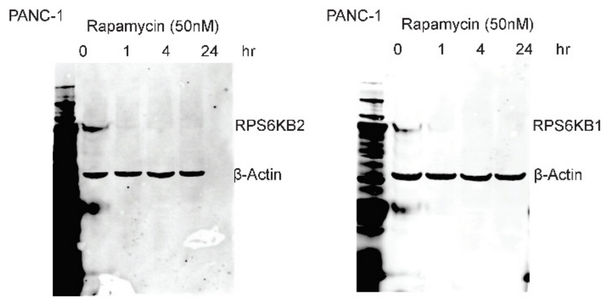

B. Full blots corresponding to Fig 2G

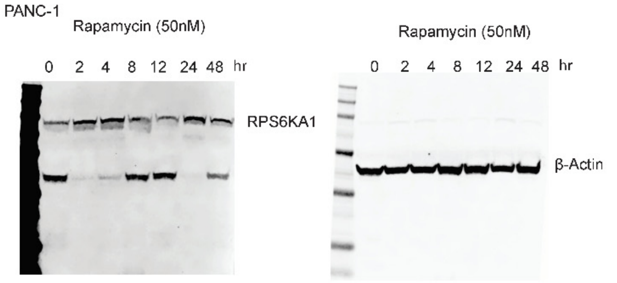

C. Full blot corresponding to Fig 2H

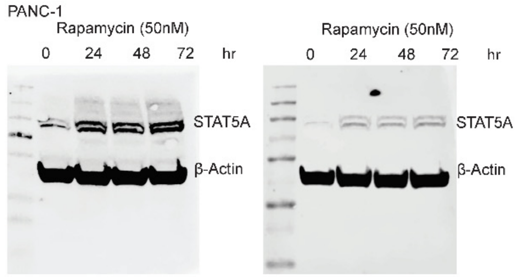

D. Full blots corresponding to Fig 4A and B

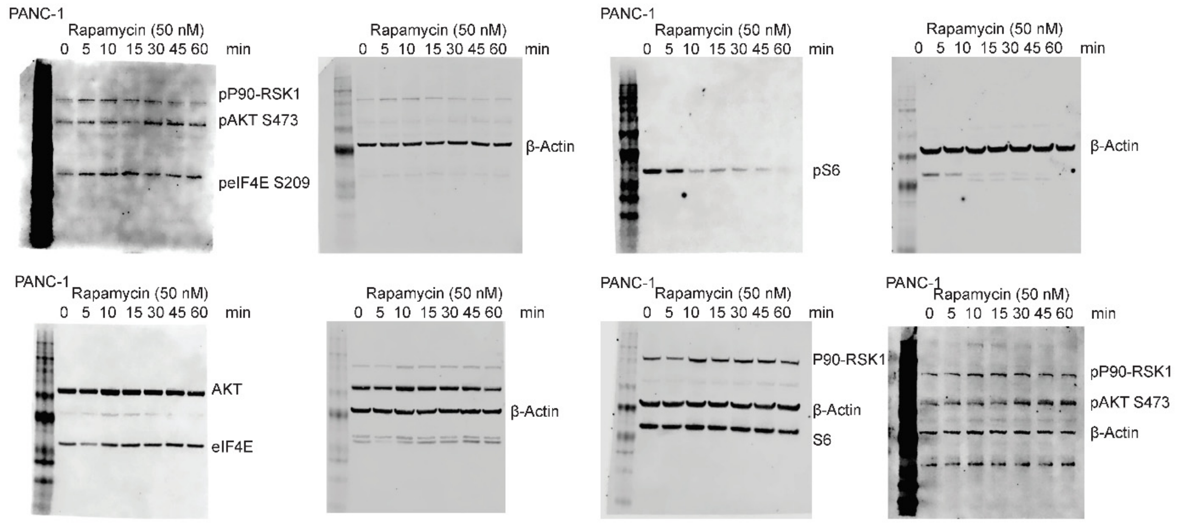

E. Full blots corresponding to Fig 4C and D

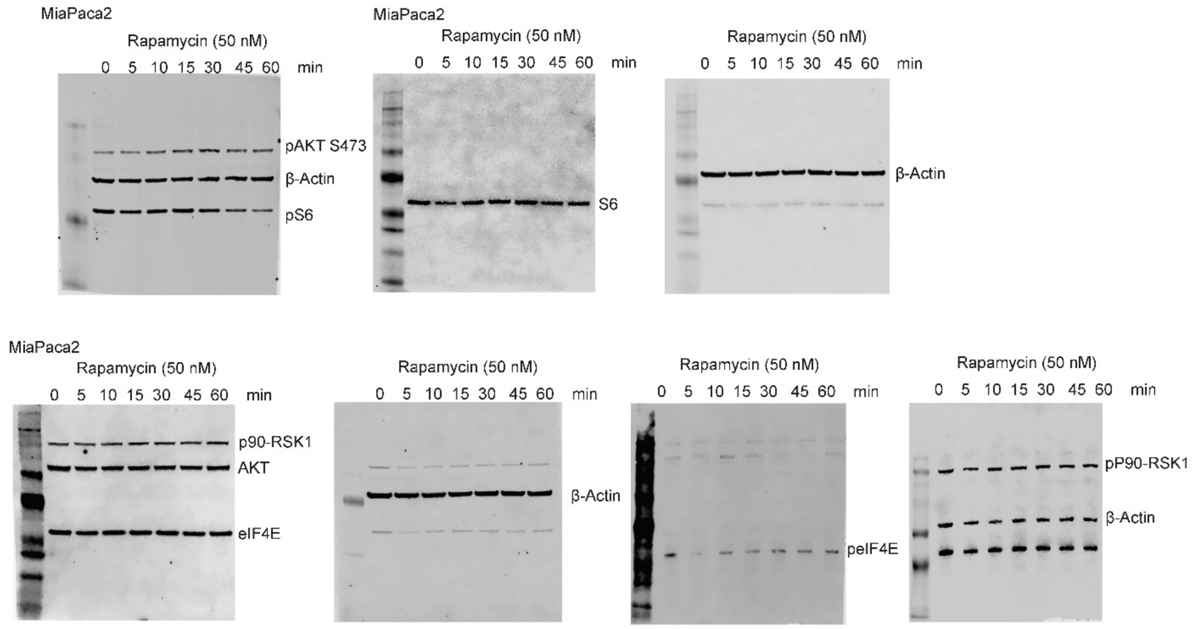

F. Full blots corresponding to Supplementary Fig 4A

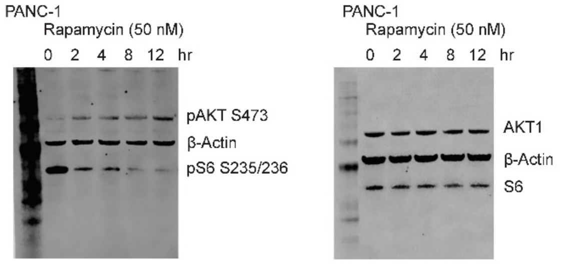

Supplementary Figure S5. Full image of immunoblots corresponding to Figures 2-4.
